# The characteristics of gut microbiota and commensal Enterobacteriaceae isolates in tree shrew (*Tupaia belangeri*)

**DOI:** 10.1186/s12866-019-1581-9

**Published:** 2019-09-02

**Authors:** Wenpeng Gu, Pinfen Tong, Chenxiu Liu, Wenguang Wang, Caixia Lu, Yuanyuan Han, Xiaomei Sun, De Xuan Kuang, Na Li, Jiejie Dai

**Affiliations:** 1 Center of Tree Shrew Germplasm Resources, Institute of Medical Biology, Chinese Academy of Medical Sciences and Peking Union Medical College, Yunnan Key Laboratory of Vaccine Research and Development on Severe Infectious Diseases, Yunnan Innovation Team of Standardization and Application Research in Tree Shrew, Zhao zong Road 66, Kunming, 650118 China; 2 Department of Acute Infectious Diseases Control and Prevention, Yunnan Provincial Centre for Disease Control and Prevention, Kunming, 650022 China

**Keywords:** Tree shrew, Gut microbiota, Commensal Enterobacteriaceae, Susceptible age groups, *Proteus* spp.

## Abstract

**Background:**

Tree shrew is a novel laboratory animal with specific characters for human disease researches in recent years. However, little is known about its characteristics of gut microbial community and intestinal commensal bacteria. In this study, 16S rRNA sequencing method was used to illustrate the gut microbiota structure and commensal Enterobacteriaceae bacteria were isolated to demonstrate their features.

**Results:**

The results showed Epsilonbacteraeota (30%), Proteobacteria (25%), Firmicutes (19%), Fusobacteria (13%), and Bacteroidetes (8%) were the most abundant phyla in the gut of tree shrew. Campylobacteria, Campylobacterales, Helicobacteraceae and *Helicobacter* were the predominant abundance for class, order, family and genus levels respectively. The alpha diversity analysis showed statistical significance (*P* < 0.05) for operational taxonomic units (OTUs), the richness estimates, and diversity indices for age groups of tree shrew. Beta diversity revealed the significant difference (P < 0.05) between age groups, which showed high abundance of Epsilonbacteraeota and Spirochaetes in infant group, Proteobacteria in young group, Fusobacteria in middle group, and Firmicutes in senile group. The diversity of microbial community was increased followed by the aging process of this animal. 16S rRNA gene functional prediction indicated that highly hot spots for infectious diseases, and neurodegenerative diseases in low age group of tree shrew (infant and young). The most isolated commensal Enterobacteriaceae bacteria from tree shrew were *Proteus* spp. (67%) and *Escherichia coli* (25%). Among these strains, the antibiotic resistant isolates were commonly found, and pulsed-field gel electrophoresis (PFGE) results of *Proteus* spp. indicated a high degree of similarity between isolates in the same age group, which was not observed for other bacteria.

**Conclusions:**

In general, this study made understandings of the gut community structure and diversity of tree shrew.

**Electronic supplementary material:**

The online version of this article (10.1186/s12866-019-1581-9) contains supplementary material, which is available to authorized users.

## Background

The tree shrew (*Tupaia belangeri*) is a small mammal similar in appearance to squirrel, widely distributed in South Asia, Southeast Asia and Southwest China [1]. Due to several specific characteristics, such as small adult body size, short reproductive and life cycle, low cost of maintenance, high brain-to-body mass ratio, and close affinity to primates, the tree shrew has been proposed as an alternative laboratory animal (nonhuman primate) in biomedical researches in recent years [2–4]. Currently, several studies have used this animal for human disease investigations, including hepatitis C virus [5], and Epstein-Barr virus [6], as well as brain development and aging [7, 8], social stress and depression [9, 10]. Although the biochemical metabolism, physiological function and genomic signature of tree shrew have been reported before [11–13], some important biological features are still unknown, for instance, the gut microbiota and commensal intestinal bacteria of this laboratory animal.

The gut microbial mutualisms, commensalisms, and pathogen interactions have been considered as important factors for animal health [14, 15]. The composition and diversity of microbial community within and between host individuals are influenced by diet, life style, and disease [16, 17]. Furthermore, previous studies showed that characteristics of the host, such as gender or age, were responsible for variation in the gastrointestinal microbiome [18, 19]. Up to present, a large number of studies on gut metagenomics by using next generation sequencing have been reported, including human, domestic or wild animals [15, 20], seldom referred to laboratory animal, especially for tree shrew. On account of its unified diet or life cycle in the laboratory feeding environment, the gut microbiota changes maybe more related with gender or age features of tree shrew. In addition, Enterobacteriaceae are the large Gram-negative bacteria, comprised of over 50 genera and 210 species. The members of this family are widely distributed across different ecological niches, including the environment, plants, and animals [21]. The majority of Enterobacteriaceae in the gut are considered commensals, as they perform beneficial for the host; however, some are considered as important pathogens in the setting of public health, such as pathogenic *Escherichia*, *Salmonella* spp., *Yersinia* spp. and *Shigella* spp. [21]. So far, there is no systemic research on intestinal commensal bacteria in tree shrew, specifically for Enterobacteriaceae*.* The identification of commensals or pathogenic bacteria from the gastrointestinal tract of this animal will provide the baseline for future human diarrhea disease researches. In this study, 16S rRNA-targeted amplicon sequencing method was used to investigate the gut microbiota of tree shrew, and Enterobacteriaceae strains were isolated to identify the characteristics of bacteria.

## Results

### Taxonomic of the tree shrew gut microbiota

**Fig. 1:**
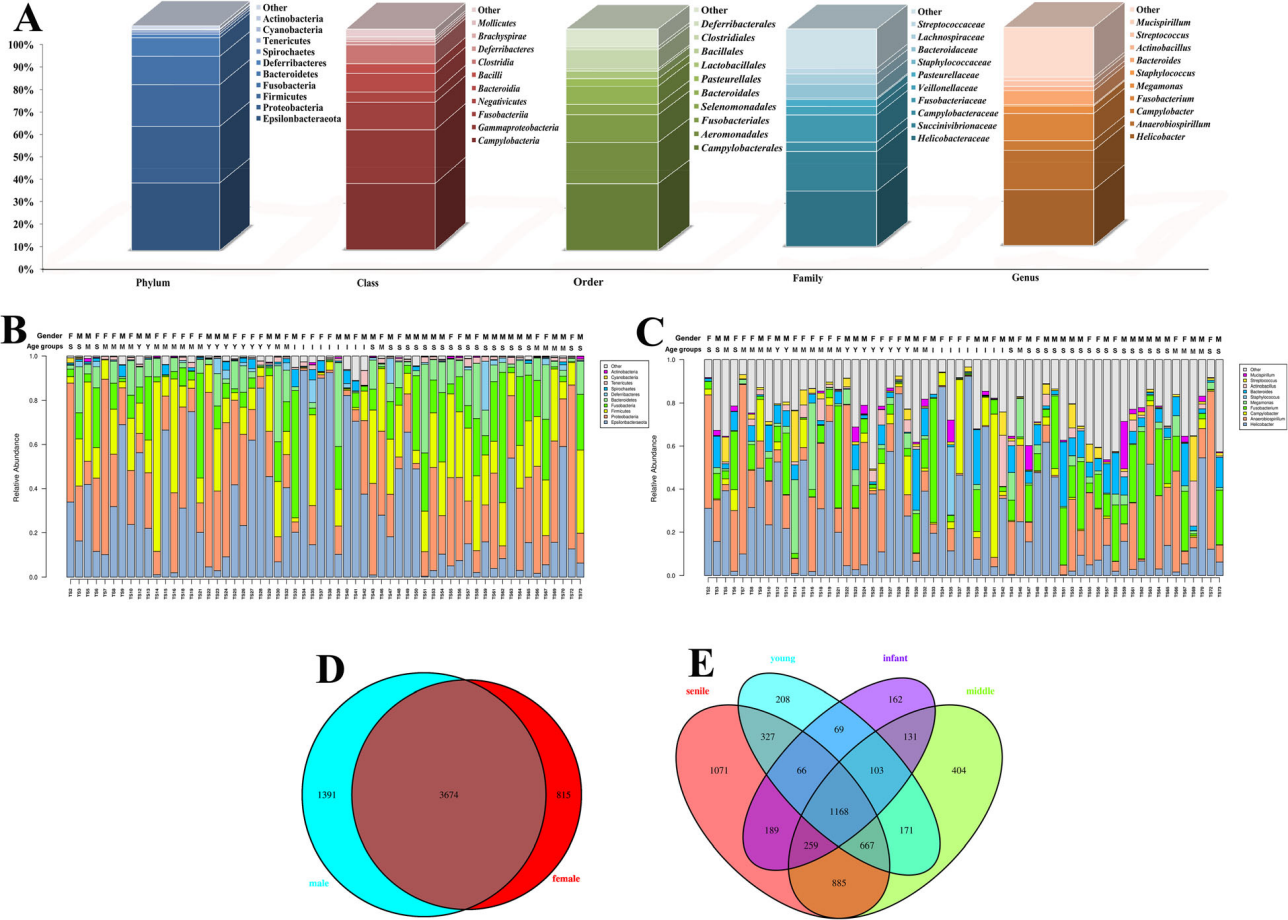
The characteristics of gut microbiota composition for tree shrew. **a** The constituent ratios of microbial communities at five levels. **b** Relative abundance of all samples at phylum level. Age groups: I (infant), Y (young), M (middle) and S (senile). Gender: F (female), M (male). **c** Relative abundance of all samples at genus level. Age groups: I (infant), Y (young), M (middle) and S (senile). Gender: F (female), M (male). **d** Venn diagram showing different OTUs between gender groups. **e** Venn diagram showing different OTUs between age groups

For the 60 fecal samples, 4,167,908 reads were obtained from tree shrew, and 4,057,554 valid reads were acquired after merging and quality trimming. The average length of amplicon was 407.52 ± 4.19 nt, ranged from 401 to 420 nt. The Q30 of sequencing was above 95% for all the samples, and GC% was 50.78% ± 0.85%. In total, 5880 OTUs were found in the database, Epsilonbacteraeota (30%), Proteobacteria (25%), Firmicutes (19%), Fusobacteria (13%), and Bacteroidetes (8%) were the most abundant bacterial communities at the phylum level; at the class level, Campylobacteria (30%), Gammaproteobacteria (24%), Fusobacteriia (13%), Clostridia (9%) and Bacteroidia (8%) were the major microbiota; Campylobacterales (30%), Aeromonadales (19%), Fusobacteriales (13%), Clostridiales (9%) and Bacteroidales (8%) were the top five at order level; the top five at family level were Helicobacteraceae (25%), Succinivibrionaceae (18%), Fusobacteriaceae (13%), Bacteroidaceae (6%), and Lachnospiraceae (5%); finally, *Helicobacter* (25%), *Anaerobiospirillum* (18%), *Fusobacterium* (13%), *Bacteroides* (6%), and *Campylobacter* (4%) were the primary microbial communities at genus level, as shown in Fig. 1a. However, the relative abundance of gut microbiota for each sample was quite different, as shown in Fig. 1b and C. The higher relative abundance of Epsilonbacteraeota were found in sample tree shrew 28 (TS28), TS34, TS37, TS38 and TS40, but lower in TS7, TS22, TS23, TS64 and TS66; TS7, TS22 and TS70 had higher abundance for Proteobacteria, but lower for TS37, TS38 and TS40. At the genus level, similar results were identified, such as TS19, TS28 and TS38 rich in *Helicobacter*; TS7, TS22, and TS72 in *Anaerobiospirillum.* According to the gender of the tree shrew, 5065 OTUs were obtained in male group, compared with 4489 in female, and more OTUs were found in male group (Fig. 1d). For the age groups, the numbers of OTUs were increased with the aging of tree shrew, as shown in Fig. 1e. One hundred and sixty two unique OTUs were discovered in infant group, 208 in young, 404 in middle and 1071 in senile group.

### Diversity analysis

The alpha diversity estimations showed that numbers of OTUs, Chao1, Shannon entropy were statistical different (*P* < 0.05) for age groups of tree shrew, indicated the significant diversity between four age groups. The OTU numbers and diversity were increased followed by the aging process. However, no statistics (*P* > 0.05) were found for gender groups of all the alpha diversity indexes except the numbers of OTUs in this animal (Table 1).

**Table 1:** The alpha diversity estimation of sequencing results in this study

Variables	Groups	Indexes (mean ± STD)			
		Numbers of OTUs	Chao1	Shannon entropy	Simpson’s index
Gender	Male	763.90 ± 183.57	1415.49 ± 358.88	4.25 ± 0.95	0.85 ± 0.11
	Female	658.03 ± 217.81	1290.31 ± 412.13	3.97 ± 1.18	0.78 ± 0.16
T-test		2.04	1.96	1.85	1.91
P value		0.046	0.058	0.079	0.061
Age groups	Infant	477.44 ± 224.85^a^	828.44 ± 375.96^a^	3.19 ± 1.23^b^	0.72 ± 0.19
	Young	697.10 ± 147.22	1263.44 ± 280.52	4.10 ± 1.01	0.80 ± 0.17
	Middle	719.78 ± 184.83	1318.21 ± 356.67	4.19 ± 1.05	0.83 ± 0.12
	Senile	806.09 ± 168.54	1503.51 ± 330.79	4.57 ± 0.93	0.85 ± 0.11
F (ANOVA)		7.26	8.65	3.91	2.04
P value		0.000	0.000	0.013	0.118

^a^the numbers of OTUs and Chao1 indexes of infant group had statistical significance (*P <* 0.05) with both young, middle and senile groups. No significant difference was found between young, middle and senile groups.

^b^Shannon entropy index of infant group had statistical significance (*P* < 0.05) with middle and senile group, but no significant difference were found between young, middle and senile groups

**Fig. 2:**
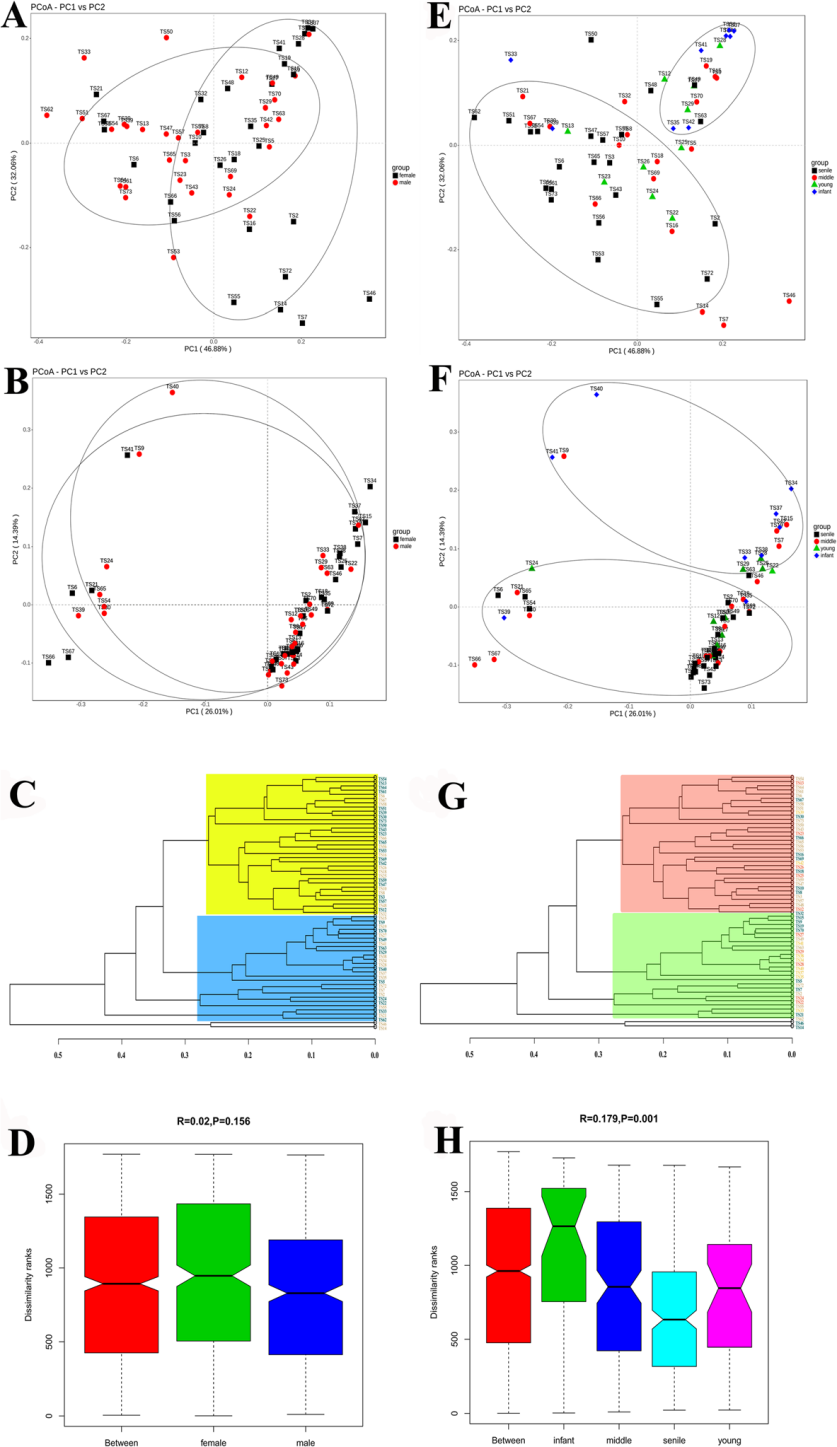
Beta diversity analysis of tree shrew fecal samples. **a** PCoA plot based on weighted unifrac distance of the male and female group. **b** PCoA plot based on unweighted unifrac distance of the male and female group. **c** UPGMA dendrogram of gender groups for all the samples. Blue samples indicated male, and pink represented female. **d** Anosim analysis between male and female group. **e** PCoA plot based on weighted unifrac distance of the four age groups. **f** PCoA plot based on unweighted unifrac distance of the four age groups. **g** UPGMA dendrogram of age groups for all the samples. Yellow samples were infant group; reds were young group; blues were middle group; pinks were senile group. **h** Anosim analysis between infant, young, middle and senile group

Beta diversity analyses were performed according to gender and age grouping. PCoA plot based on weighted and unweighted Fast UniFrac distance metric revealed two clustering gender groups were generated; however, large numbers of male and female samples were mixed together and cross connected from two cycles shown in Fig. 2a and b. The UPGMA dendrogram of gender groups showed two clusters in Fig. 2c (yellow and blue areas); each cluster also contained mix male and female tree shrew samples. The Anosim analysis indicated no statistical significance between male and female groups (R = 0.02, *P* = 0.156), as shown in Fig. 2d. For the age groups of tree shrew, two obvious clustering groups were found between infant and senile (Fig. 2e and f), while the young and middle were randomized distributed in PCoA plot. The UPGMA dendrogram of age groups also showed two clusters (Fig. 2g); the majority of red cluster area was senile group samples, parts of the middle group samples were located in this cluster, such as TS67, TS30, and TS66. The green cluster area in Fig. 2g contained most infant group samples, young and middle group samples were found in this cluster as well. The Anosim statistic revealed significant difference among age groups (R = 0.179, *P* = 0.001), as shown in Fig. 2h.

### Variation analysis

**Fig. 3:**
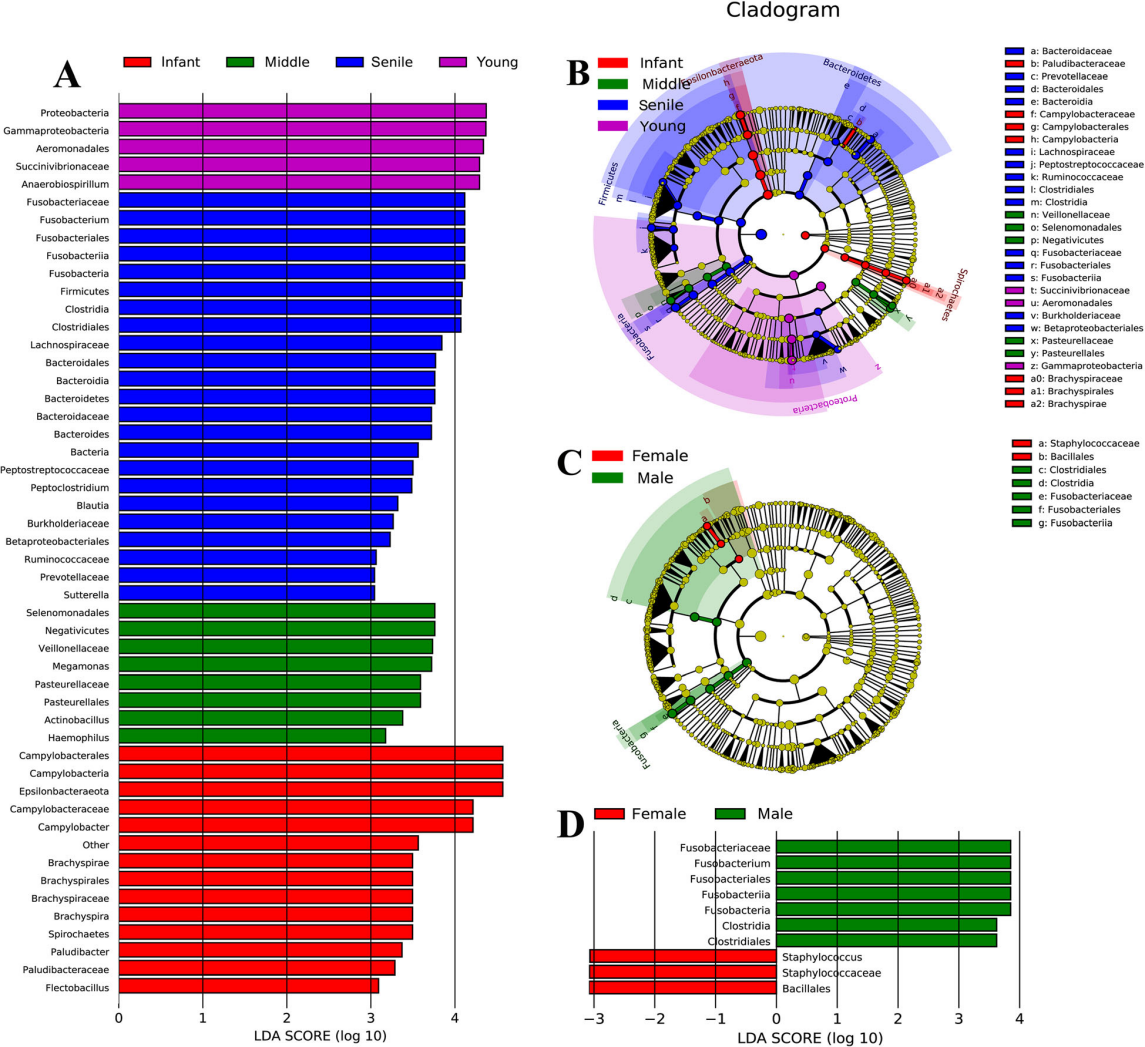
Indicator microbial groups and phylogenetic distribution of microbial communities between age and gender groups for tree shrew. **a** Indicator microbial groups in the four age group samples with LDA scores higher than 3.0. **b** Cladogram indicated the phylogenetic distribution of microbial communities associated with four age groups; lineages with LDA values of 3.0 or higher as determined by LEfSe were shown. Differences were represented by the color of the most abundant class. Red indicated infant group, purple young group, green middle group, and blue senile group; yellow represented insignificant difference. **c** Cladogram indicated the phylogenetic distribution of microbial communities associated with gender groups; lineages with LDA values of 3.0 or higher as determined by LEfSe were shown. Differences were represented by the color of the most abundant class. Red indicated female group; green represented male group. **d** Indicator microbial groups in the gender group samples with LDA scores higher than 3.0

LEfSe analyses were performed on significant differences of microbial taxa in abundance among tree shrew age and gender groups. The LDA score ≥ 3.0 of gut microbiota between four age groups were shown in Fig. 3a. There were thirteen microbial taxa enriched in the infant group, five microbial taxa enriched in the young group, eight microbial taxa enriched in the middle group, and twenty-three enriched in the senile group. The most different abundant microbial taxa in infant group were Epsilonbacteraeota (the phylum), Spirochaetes (the phylum), Campylobacteria (the class), Brachyspirae (the class), Campylobacterales (the order), Brachyspirales (the order), Campylobacteraceae (the family) and Brachyspiraceae (the family). Proteobacteria (the phylum), Gammaproteobacteria (the class), Aeromonadales (the order) and Succinivibrionaceae (the family) were the primary taxa in young group. Meanwhile, Negativicutes (the class), Selenomonadales (the order), Pasteurellales (the order) and Pasteurellaceae (the family) were significantly enriched in middle group. Firmicutes (the phylum), Fusobacteria (the phylum), Bacteroidetes (the phylum), Clostridia (the class), Fusobacteriia (the class), Bacteroidia (the class), Clostridiales (the order), Fusobacteriales (the order), Bacteroidales (the order), Fusobacteriaceae (the family) and Bacteroidaceae (the family) were the representative taxa in the senile group. Cladogram showed the phylogenetic distribution of dominant classified microbial taxa associated with the age groups (Fig. 3b). The biomarkers of significant differences in abundance between male and female were also showed in Fig. 3c. Cladogram indicated that Clostridia (the class), Fusobacteriia (the class), Clostridiales (the order), Fusobacteriales (the order) and Fusobacteriaceae (the family) were enriched in male group, while Bacillales (the order) and Staphylococcaceae (the family) were predominant in female group. The gut microbial taxa presented statistically significant differences with an LDA threshold ≥3.0 between male and female group were shown in Fig. 3d.

KEGG pathway annotation results based on PICRUSt revealed organismal systems, cellular process and human diseases concentrated on infant and young age groups, while the genetic information processing, metabolism and environmental information processing for senile group (Additional file 1). The metabolic pathways analysis showed higher hot spots for cell growth and death, infectious diseases, translation, genetic information processing, energy metabolism, neurodegenerative diseases, cell motility and environmental adaptation in low age group of tree shrew (infant and young); metabolism, membrane transport, endocrine system, carbohydrate metabolism, replication and repair were higher in senile age group, as Additional file 2 shown. The details of annotation information for metabolic pathway among four age groups were shown in Additional file 3. However, the annotation results according to gender groups had no such trends of concentration for gene functional predictions, as shown in Additional files 4, 5, 6.

### Characteristics of isolated Enterobacteriaceae bacteria

**Fig. 4:**
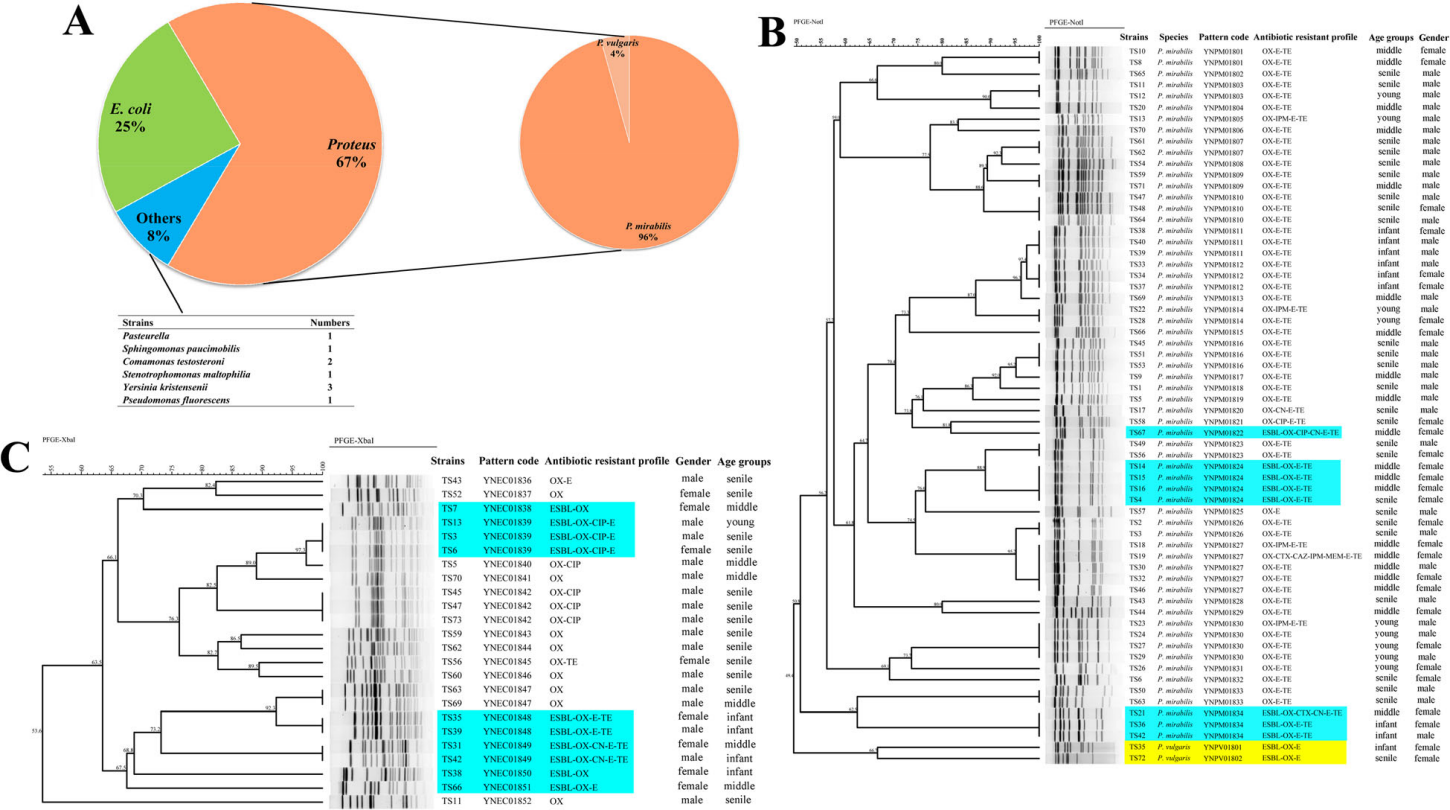
The characteristics of isolated Enterobacteriaceae bacteria from tree shrew. **a** The isolation results of fecal samples for tree shrew. **b** PFGE cluster results of 71 *Proteus* spp. in this study. The yellow area was *P. vulgaris* strains, blue area indicated ESBL isolates for *P. mirabilis.* **c** PFGE cluster results of 25 *E. coli* in this study. Blue area indicated ESBL isolates for *E. coli*

One hundred and five strains were isolated from 73 tree shrew feces; among them, 28 samples had multiple species isolated. *Proteus* spp. (71, 67%) and *E. coli* (25, 25%) were the most Enterobacteriaceae bacteria (Fig. 4a). For *Proteus* spp., the majority of species was *P. mirabilis* (68, 96%), and only three *P. vulgaris* (4%) were isolated. All the entero-pathogenic bacteria, including *Vibrio* spp., *Salmonella* spp., and *Shigella* spp. etc. were not found in this study, including diarrheogenic *E. coli* by multiplex PCR. The *Proteus* spp. isolated results had no statistical significance with gender (H = 0.01, *P* = 0.922) and age groups (H = 0.348, *P* = 0.555) of tree shrew. The antibiotic resistant results showed high resistant rate for Oxacillin (OX) (100%), Erythromycin (E) (100%) and Tetracycline (TE) (94.40%), 12 isolates (16.90%) were identified as extended-spectrum β-lactamases strains (ESBL) (Table 2). The gender of tree shrew (H = 7.774, *P* = 0.005) and species of *Proteus* spp. (H = 15.184, *P* = 0.000) showed the statistical difference with ESBL strains. 83.30% ESBL *Proteus* spp. were isolated from female tree shrew, compared with 16.70% in male group; and all the three *P. vulgaris* were the ESBL, but 13.20% for *P. mirabilis.* The Cefotaxime (CTX), Ceftazidime (CAZ), Meropenem (MEM), Ciprofloxacin (CIP), Gentamicin (CN) and TE resistant results had no statistical significance (*P* > 0.05) with gender and age groups. Thirty six PFGE patterns were identified for all the *Proteus* spp. strains, showing a high degree of polymorphism (Fig. 4b), and two species of *Proteus* spp. were divided into two cluster groups. The PFGE patterns had significant difference (H = 55.273, *P* = 0.009) with age groups of tree shrew, indicated the highly similarity between isolates in the same age group, such as YNPM01811 and YNPM01812 in infant group; YNPM01830 in young group; YNPM01824 and YNPM01827 in middle group; YNPM01810 in senile group, as shown in Fig. 4b.

**Table 2:** The antibiotics resistant results of isolated Enterobacteriaceae bacteria in this study

Antibiotics	Interpret	*Proteus* spp.		*E.coli*	
		Strain numbers	Percent (%)	Strain numbers	Percent (%)
ESBL	Resistant (R)	12	16.90%	10	40.00%
	Sensitive (S)	59	83.10%	15	60.00%
OX	R	71	100.00%	25	100.00%
	S	–	–	–	–
CTX	R	2	2.80%	–	–
	S	69	97.20%	25	100.00%
CAZ	R	1	1.40%	–	–
	S	69	97.20%	25	100.00%
	Intermediate (I)	1	1.40%	–	–
IPM	R	8	11.30%	–	–
	S	49	69.00%	25	100.00%
	I	14	19.70%	–	–
MEM	R	1	1.40%	–	–
	S	70	98.60%	25	100.00%
CIP	R	2	2.80%	8	32.00%
	S	69	97.20%	15	60.00%
	I	–	–	2	8.00%
CN	R	3	4.20%	2	8.00%
	S	68	95.80%	23	92.00%
AK	R	–	–	–	–
	S	71	100.00%	25	100.00%
E	R	71	100.00%	9	36.00%
	S	–	–	13	52.00%
	I	–	–	3	12.00%
TE	R	67	94.40%	6	24.00%
	S	4	5.60%	19	76.00%

The *E. coli* isolation results also had no statistical significance with gender (H = 3.202, *P* = 0.074) and age groups (H = 1.422, *P* = 0.233) of tree shrew. Ten ESBL *E. coli* strains (40%) were found in this study, and high antibiotic resistant rates were for OX (100%), E (36%), CIP (32%), and TE (24%) (Table 2). The gender (H = 5.765, *P* = 0.016) and age groups (H = 11.082, *P* = 0.001) showed the statistical difference with ESBL *E. coli* strains. Sixty percent of the ESBL were found in female tree shrew, and 40.0% for male. Furthermore, half of the ESBL strains were isolated from infant group. Similar results could be obtained for TE with gender (H = 4.186, *P* = 0.041) and age groups (H = 7.412, *P* = 0.006) of this laboratory animal. The CIP, CN, and E resistant results had no statistical significance (*P* > 0.05) with gender and age groups. Seventeen PFGE patterns were found for all the *E. coli* strains, showing no statistical difference (*P* > 0.05) with gender or age groups (Fig. 4c).

The antimicrobial resistant-associated genes were showed in Table 3. *TEM* and *CTX-M* were both detected for *Proteus* spp. and *E. coli* ESBL strains, among them, *TEM* was the major resistant gene. All of the *Proteus* spp. resistant to Imipenem (IPM) had *NDM1*, and most of CIP resistant gene was *qnrB* for tree shrew Enterobacteriaceae strains. The most resistant-associated genes for erythromycin of *Proteus* spp. were *ereA* (54.93%) and *ereB* (16.90%), but *tetE* (70.15%) and *tetO* (17.91%) for tetracycline (Table 3).

**Table 3:** The antimicrobial resistant-associated genes detected in this study

Resistant antibiotic	Genes	*Proteus* spp.		*E. coli*	
		Numbers	Percent (%)	Numbers	Percent (%)
ESBL	*TEM*	5	41.67%	8	80.00%
	*CTX-M*	3	25.00%	2	20.00%
	Unknown	4	33.33%	–	–
CTX and CAZ	Unknown	2	100.00%	–	–
IPM	*NDM1*	8	100.00%	–	–
CIP	*qnrA*	–	–	3	37.50%
	*qnrB*	2	100.00%	4	50.00%
	*qnrS*	–	–	1	12.50%
CN	*aadA1*	1	33.33%	–	–
	*aacA4*	2	66.67%	2	100.00%
E	*ereA*	39	54.93%	1	11.11%
	*ereB*	12	16.90%	3	33.33%
	*mphA*	4	5.63%	3	33.33%
	Unknown	16	22.54%	2	22.23%
TE	*tetA*	3	4.48%	6	100.00%
	*tetB*	5	7.46%	–	–
	*tetE*	47	70.15%	–	–
	*tetO*	12	17.91%	–	–

## Discussion

Comparative genome analysis between tree shrew and human revealed that there was a high sequence identity for genes/pathways involved in infectious diseases and neuropsychiatric disorders [11]. The proteomics of muscle and liver tissues for tree shrew indicated that almost half of the proteins were highly similar to those of human [22]. Besides, at the neurophysiological or neuroanatomical levels, a close homology between tree shrew and human in the area of visual cortex was also found [23]. All these pieces of evidence have laid the foundation for using the tree shrew to study human related diseases [24]. Therefore, study on the characteristics of tree shrew gut microbiota could provide us the better understandings of the baseline for tree shrew used as a laboratory model.

The mammalian intestinal tract has large numbers of bacteria, playing an important role in host metabolism, immunity, nutrition, and behaviors. The numbers of microorganisms in the gut exceed the host cells and the functions encoded by gut microbiota greatly surpass those of the host [25]. Since the widely use of next generation sequencing techniques, several studies have substantially increased our knowledge for both host-associated and environmental microbial communities. Previous study [20] showed gut microbiota at higher taxonomic levels among 60 mammalian species, and most referred to Firmicutes (65.7%) and Bacteroidetes (16.3%), dominated out of 75 known microbial phyla. These phyla were originally shown to compose the majority of sampled human gut-associated phylotypes. The other phyla represented were the Proteobacteria (8.8%), Actinobacteria (4.7%), and Verrucomicrobia (2.2%) etc. These results were in accordance with that the ancestor of amniotes possessed the microbiomes mostly comprised Firmicutes and Bacteroidetes [26]. Our previous research on migrated gulls also indicated Firmicutes and Proteobacteria were the most abundance phyla of this wild animal [27]. However, in this study, Epsilonbacteraeota was the most abundance phylum level for tree shrew, followed by Proteobacteria, and Firmicutes; furthermore, the *Helicobacter* and *Campylobacter* were the important microbial communities at genus level, belonged to Epsilonbacteraeota. In the past, Epsilonproteobacteria was the fifth validly described class of the phylum Proteobacteria. Waite et al. [28] reassigned this class to a novel phylum for propose the name Epsilonbacteraeota (phyl. nov.) based on assessment of nearly 300 phylogenetic tree topologies in 2017. It was very interesting that Epsilonbacteraeota was the most gut microbiota for tree shrew, especially for infant group. Some studies reported *Helicobacter* and *Campylobacter* species colonized the intestinal tract of many domestic animals, and zoo mammals. Goto et al. [29] found the current status of *Helicobacter* contamination in laboratory mice, rats, gerbils, and house musk shrews, the 66.7% colonies of *H. suncus* were detected in shrew. Whary et al. [30] revealed the naturally acquired *Helicobacter* infections in commonly used laboratory rodent species, including mice, rats, gerbils, and hamsters. Consequently, it was not surprised that Epsilonbacteraeota was the majority of microbial community for tree shrew; however, the results of *Helicobacter* infection of laboratory animals should be paid attention for further animal model investigation.

Several studies have exemplified the role that the intestinal microbe played in mammalian physiology, human health and disease [15, 17]. The lack of balanced and healthy gut microbiota has been linked to susceptibility to infection, decreased lymphocyte and intestinal macrophage proliferation of the hosts [31]. However, these associations have not to be investigated in depth in nonhuman primates. The nonhuman primates were the most biologically related research animal models for human, and a better understanding of the gut microbial communities would provide the opportunity to evaluate the influence in nonhuman primate evolution and ecology [25]. To date, seldom studies referred to the relationship between gender and age with gut microbiota composition. In our study, we found the aging of tree shrew was significantly responsible for variation of the microbial communities; the gut microbiota diversity was increased followed by the aging process of this laboratory animal, and for each age group, there were some representative bacteria. Amato et al. [18] determined that adult males, adult females, and juveniles have distinct microbiome compositions of black howler monkey, and juvenile and adult howlers possibly obtained nutritional benefits from the intestinal microbiome for their growth and reproduction. Ren et al. [19] found that wild yellow baboons possessed two different microbiome configurations, and determined that host age, diet and rainfall, were largely responsible for variation in the gastrointestinal microbiome. The tree shrew used in this study was closed population, the diet and feeding conditions were identical for the entire animal. Therefore, we considered that aging process was really responsible for variation of gut microbiota for tree shrew.

None of the intestinal pathogenic bacteria was detected in this study, among them; Enterobacteriaceae was the most one, especially for *Proteus* spp. and *E. coli*. Gordon et al. [32] analyzed 642 mammalian hosts for their isolated Enterobacteriaceae bacteria in Australia, and their results showed *E. coli* was the most common of the 24 enteric species. In our study, few species of Enterobacteriaceae bacteria was isolated compared with the wild migrated bird of our previous research [27], which possibly due to the single feeding environment or life cycle of tree shrew. Many wild and domestic animals, such as mammals, birds, reptiles, and insects were the hosts of *Proteus* spp. bacteria [33]. The relations between *Proteus* spp. with their hosts were still sometimes not determined. In our study, the isolated *Proteus* spp. was more likely to be the commensal, since no diseases or symptoms appeared in all these tree shrews. However, the antimicrobial resistant Enterobacteriaceae bacteria and related genes were detected among these strains, especially for erythromycin, tetracycline and β-lactamase, indicated the highly antibiotic resistant isolates were commonly existed in this laboratory animal.

## Conclusions

As an alternative laboratory animal, tree shrew became widely used for human disease studies recently. In this study, we analyzed the gut microbiota structure and commensal Enterobacteriaceae bacteria for tree shrew. Significant diversity of microbial community was found between each sample, and the diversity was increased followed by the aging of this laboratory animal. The most isolated commensal Enterobacteriaceae bacteria were *Proteus* spp. and *E. coli*. Among these strains, the antibiotic resistant isolates were commonly found. In general, this study made understandings of the gut community structure and diversity of tree shrew.

## Methods

### Sample collections and DNA extraction

Seventy-three tree shrew fecal samples were collected at the Center of Tree Shrew Germplasm Resources, Institute of Medical Biology, Chinese Academy of Medical Science and Peking Union Medical College in Kunming, China. The tree shrews were closed population, and healthy without visible signs of tumors or disease, 39 were male, and 34 were female. The average age was 35.55 ± 22.76 months, ranged from 2 months to 75 months. We divided these animals into four age groups according to the previous study with some modification [1, 12]; the infant group was under 7 months, young group was aged between 8 to 18 months, middle group was 19 to 42 months, and over 43 months was defined as senile. All of the tree shrews used in this study were the first filial generation, weighing 138.67 ± 20.36 g. Each tree shrew was housed in independent sterilized stainless steel cage containing hygienic food and water. The commercial full-price nutritive pellet was used for feeding twice a day, and the clean apple was fed once a week. Fresh fecal samples were collected and stored at − 80 °C until processing all samples together for gut microbiota analysis. Each fecal sample was handled for two ways, one was isolated the commensal Enterobacteriaceae bacteria by using fresh feces, another was selected 60 samples to extract the genomic DNA for 16S rRNA sequencing. The total genomic DNA was extracted by using fecal sample’s DNA extraction kit (Tiangen, Beijing) following the manufacturer’s instructions. All the DNA samples were stored at − 20 °C until usage.

### PCR amplication, library construction and sequencing

The 16S rRNA gene ranged from V3 to V4 variable region was used as the target for bacterial community investigation by Illumina Miseq sequencing. PCR amplication primer was used according to Klindworth et al. [34] study and the protocal of library preparation guideline of Illumina. In general, PCR was performed by using KAPA HiFi HotStart ReadyMix kit (Kapa, Biosystems). Each PCR reaction contained genomic DNA 2.5 μl, forward and reverse primers 5 μl respectively, and KAPA mixture 12.5 μl. The amplication procedure was based on our previous study, and then the products were purified with AMPure XP magnetic beads (Beckman, Coulter), quantified using Qubit fluorometer (Invitrogen, Life Technologies). The secondary PCR amplication was performed to add the Illumina Nextera barcodes, using i5 and i7 primers following the manufacturer’s instruction, and then the purification process was executed again to remove nontarget fragments. Finally, the amplicons were normalized, pooled and sequencing was conducted using Illumina Miseq sequencing system (Illumina, SanDiego, USA).

### Bioinformatics and statistics

The raw data were trimmed for quality check and filtered of low quality (<Q25) reads. The paired end reads were merged to generate tags by using CLC Genomics Workbench 9.5.2 (QIAGEN, Denmark) [27]. The combinations of software QIIME (version 2) [35], USEARCH (version 11) [36] and R package (version 3.2) [37] were used for bioinformatics analysis. The merged tags were filtered by QIIME, and all the sequences were clustered into operational taxonomic units (OTUs), according to 97% sequence similarity against Silva 132 database [38] using the UPARSE pipeline (http://drive5.com/usearch/manual/uparsecmds). OTUs were named based on the genus level using SILVA taxonomic nomenclature.

Principal co-ordinates analysis (PCoA) was performed to visualize the similarities between samples for gender and age groups according to Bray-Curtis using Ape package. Anosim (Analysis of similarities) was used to compare the microbial composition difference between groups, and the statistical significant group (*P* < 0.05) was analyzed by LEfSe (Linear discriminant analysis Effect Size) to identify the biomarker bacteria between groups (*P* value cutoffs, 0.05). PICRUSt (Phylogenetic investigation of communities by reconstruction of unobserved states) [39] was used to predict the functional contents from 16S rRNA gene through KEGG pathway database. Statistical analysis was performed by using SPSS software package (version 16.0, IBM, USA). Kolmogorov-Smirnov, T-test, ANOVA or Kruskal-Wallis H test were used if appropriate. P value of < 0.05 was recognized as statistical significance. Sequence data were deposited on the NCBI database by the SRA accession: SRP151653.

### Isolation of Enterobacteriaceae bacteria

The intestinal Enterobacteriaceae bacteria were isolated based on previous study [27]. All the fecal samples were inoculated on MacConkey Agar and Xylose Lysine Desoxycholate (XLD) agar (Luqiao, Beijing), incubated at 37 °C for 24 h. Selenite Brilliant Green Broth (SBG) and Buffered Peptone Water (BPW) (Luqiao, Beijing) were used as enrichment broth to isolate the *Salmonella* spp. and *Vibrio* spp., then the enrichments were inoculated on Salmonella Shigella agar (SS) and Thiosulfate citrate bile salts sucrose agar (TCBS) (Luqiao, Beijing), incubated at 37 °C for 24 h. *Yersinia* spp. was isolated according to wang et al. [40] method. All the suspected Enterobacteriaceae bacteria were picked and identified by using Vitek Compact 2 biochemical identification system (bioMérieux). In addition, all the isolated *E. coli* were detected using multiplex PCR diagnostic kit (ABTechnology, Beijing) for the diarrheogenic *E. coli* (DEC). The workflow for Enterobacteriaceae isolation and identification in this study was shown in Additional file 7.

### Antibiotic resistant test and genes detection

All the isolates were performed antibiotic resistant test by broth micro-dilution method using customized microtiter plates (Sensititre, UK) according to the manufacturers’ instructions. The minimum inhibitory concentrations (MICs) for 12 antibiotics was determined, Amoxicillin (AML), Amoxicillin/Clavulanic acid (AMC), Oxacillin (OX), Cefotaxime (CTX), Ceftazidime (CAZ), Imipenem (IPM), Meropenem (MEM), Ciprofloxacin (CIP), Gentamicin (CN), Amikacin (AK), Erythromycin (E), and Tetracycline (TE). The tests were interpreted in accordance with the Clinical and Laboratory Standards Institute (CLSI) guidelines (M100-S25, 2015); *E. coli* ATCC 25922 was used as quality control. The breakpoints of MIC values for Enterobacteriaceae bacteria were shown in Additional file 8. Antimicrobial resistant-associated genes were detected by PCR and sequenced using the primers based on previous studies [41–45]. The primers for different resistant genes were shown in Additional file 9. The bacterial genomic DNA was extracted by bacteria genomic DNA extraction kit (Tiangen, Beijing). The PCR reaction was performed in 20 μl volume, contained 10 μl Premix Taq (TaKaRa, Japan), 8 μl water, 0.5 μl each primers, and 1 μl sample DNA. The amplification procedures were 94 °C 5 min, followed by 30 cycles: 94 °C 15 s, 55 °C 30 s, 72 °C 30 s, and finally 72 °C 10 min. The amplified products were detected in 1.5% agarose gel. The positive amplicons were sent for bidirectional sequencing by TaKaRa, Japan.

### Pulsed-field gel electrophoresis (PFGE)

PFGE was performed for isolated *Proteus* spp. and *E. coli* strains according to previous researches [46, 47], each plug was digested with *NotI* (TaKaRa, Japan) for *Proteus* spp. and *XbaI* (TaKaRa, Japan) for *E. coli.* CHEF-Mapper (Bio-Rad, USA) was used for electrophoresis, and the pulse time ranged from 5 s to 40 s (*Proteus* spp.) and 6.76 s to 35.38 s (*E. coli*) for 19 h. The gels were stained with Gel-Red (Biotium) and visualized by using gel imaging system (Bio-Rad, Gel DocXR). PFGE patterns were analyzed using BioNumerics version 6.6, and dendrograms were constructed using the Dice coefficient and un-weighted pair group methods with the arithmetic mean algorithm (UPGMA).

## Additional files

Additional file 1: Heatmap of KEGG pathway annotation results of age groups based on PICRUSt (level 1). (PDF 792 kb)

Additional file 2: Heatmap of the metabolic pathways results of age groups (level 2). (PDF 1231 kb)

Additional file 3: Heatmap of the detailed annotation information for metabolic pathway among four age groups (level 3). (PDF 1096 kb)

Additional file 4: Heatmap of KEGG pathway annotation results of gender groups based on PICRUSt (level 1). (PDF 795 kb)

Additional file 5: Heatmap of the metabolic pathways results of gender groups (level 2). (PDF 1021 kb)

Additional file 6: Heatmap of the detailed annotation information for metabolic pathway among gender groups (level 3). (PDF 1016 kb)

Additional file 7: The workflow for Enterobacteriaceae isolation and identification in this study. (PDF 1115 kb)

Additional file 8: The breakpoints of MIC values for Enterobacteriaceae bacteria in this study. (PDF 102 kb)

Additional file 9: The PCR primers for different resistant genes in this study. (PDF 359 kb)

## Data Availability

The datasets generated and/or analysed during the current study are available in the NCBI database repository by the SRA accession: SRP151653, [https://www.ncbi.nlm.nih.gov/sra?linkname=bioproject_sra_all&from_uid=478287].

## Abbreviations

LEfSe: Linear discriminant analysis Effect Size
OTUs: Operational taxonomic units
PCoA: Principal co-ordinates analysis
PFGE: Pulsed field gel electrophoresis
PICRUSt: Phylogenetic investigation of communities by reconstruction of unobserved states
