# Supplementary figures and images for: The characteristics of gut microbiota and commensal Enterobacteriaceae isolates in tree shrew (Tupaia belangeri)
Source: BMC Microbiol. 2019 Sep 2;19:203. doi: 10.1186/s12866-019-1581-9 (PMC6721287; doi:10.1186/s12866-019-1581-9)

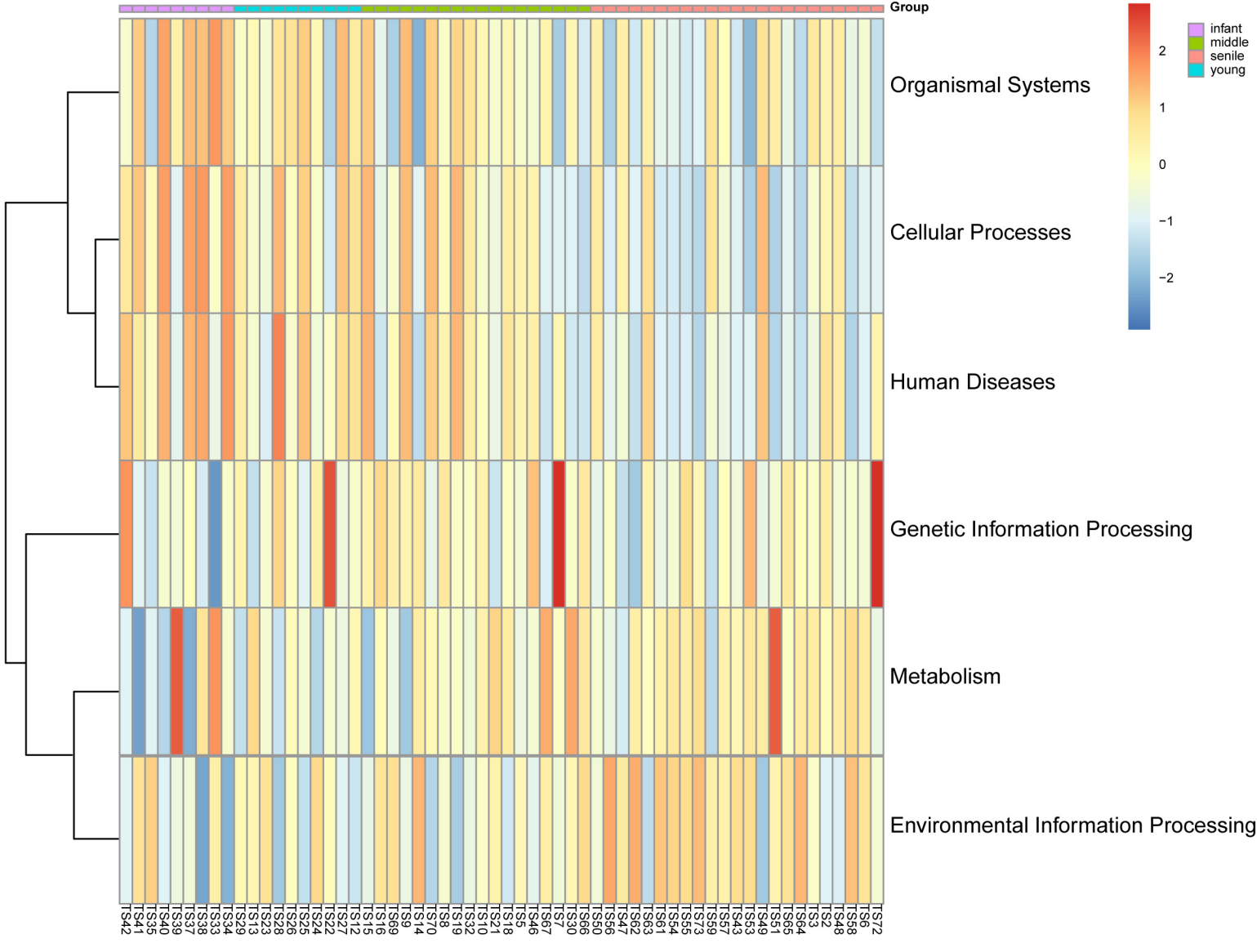

Supplement: Supplementary file 1 — Heatmap of KEGG pathway annotation results of age groups based on PICRUSt (level 1). (PDF 792 kb) [file 12866_2019_1581_MOESM1_ESM.pdf]

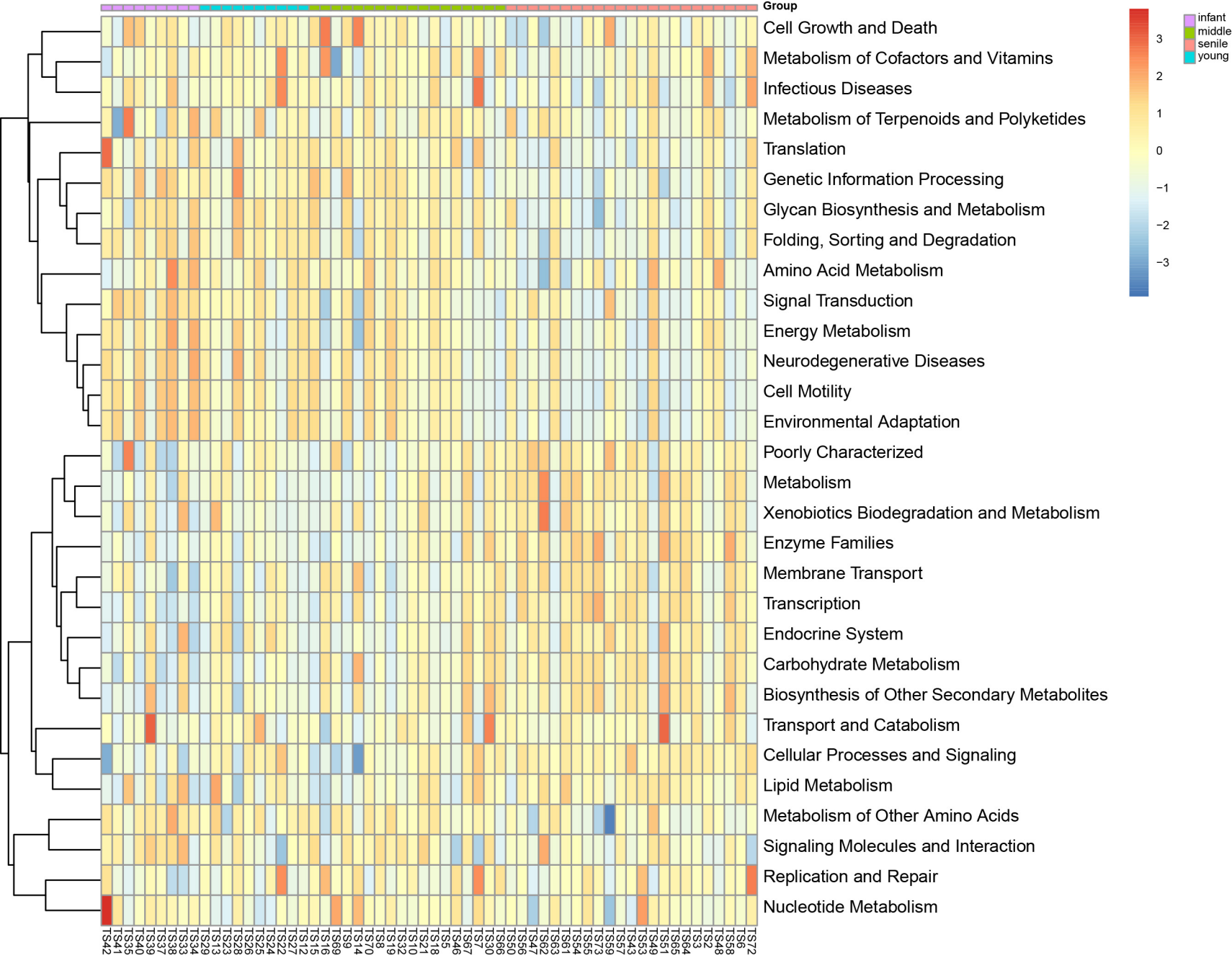

Supplement: Supplementary file 2 — Heatmap of the metabolic pathways results of age groups (level 2). (PDF 1231 kb) [file 12866_2019_1581_MOESM2_ESM.pdf]

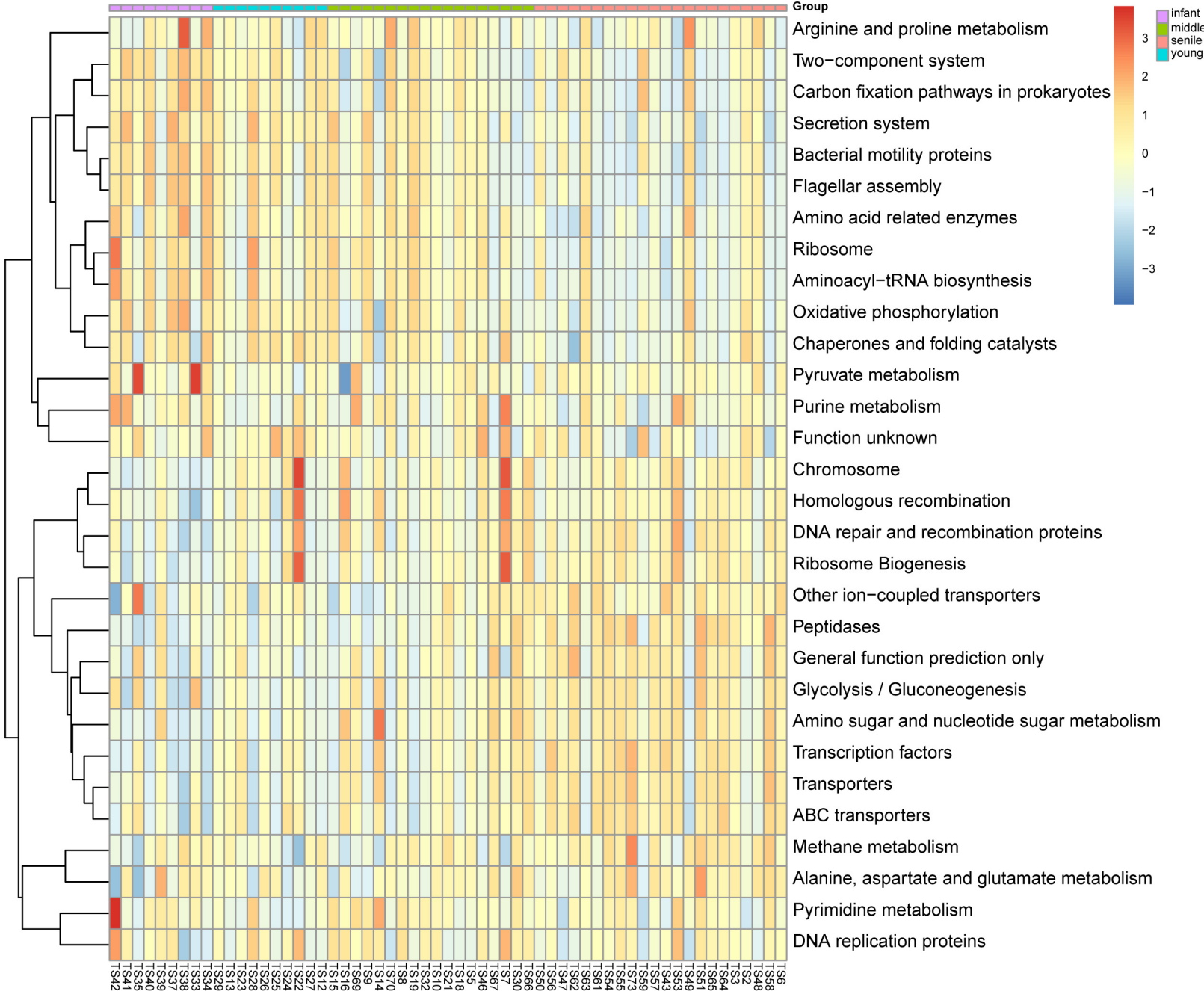

Supplement: Supplementary file 3 — Heatmap of the detailed annotation information for metabolic pathway among four age groups (level 3). (PDF 1096 kb) [file 12866_2019_1581_MOESM3_ESM.pdf]

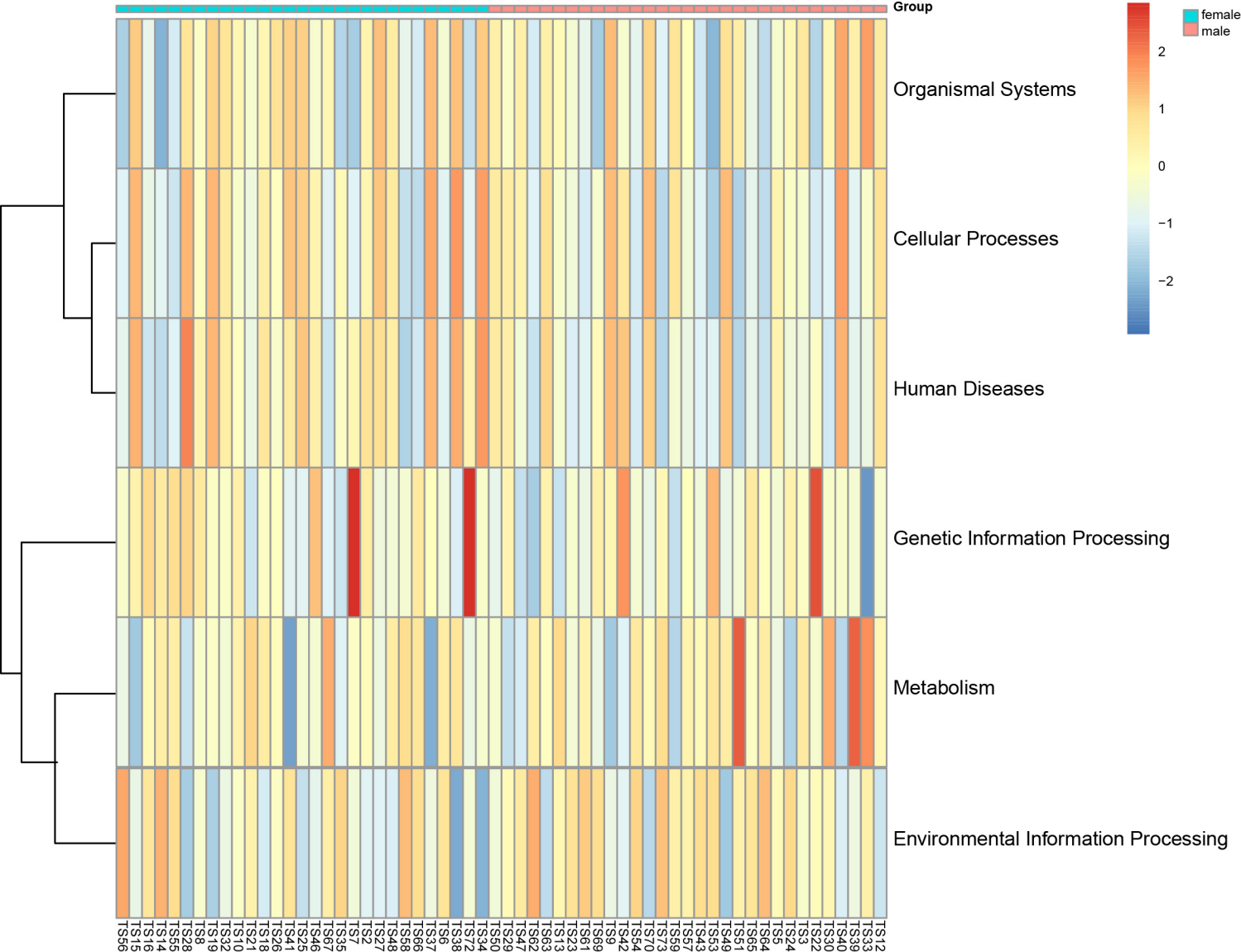

Supplement: Supplementary file 4 — Heatmap of KEGG pathway annotation results of gender groups based on PICRUSt (level 1). (PDF 795 kb) [file 12866_2019_1581_MOESM4_ESM.pdf]

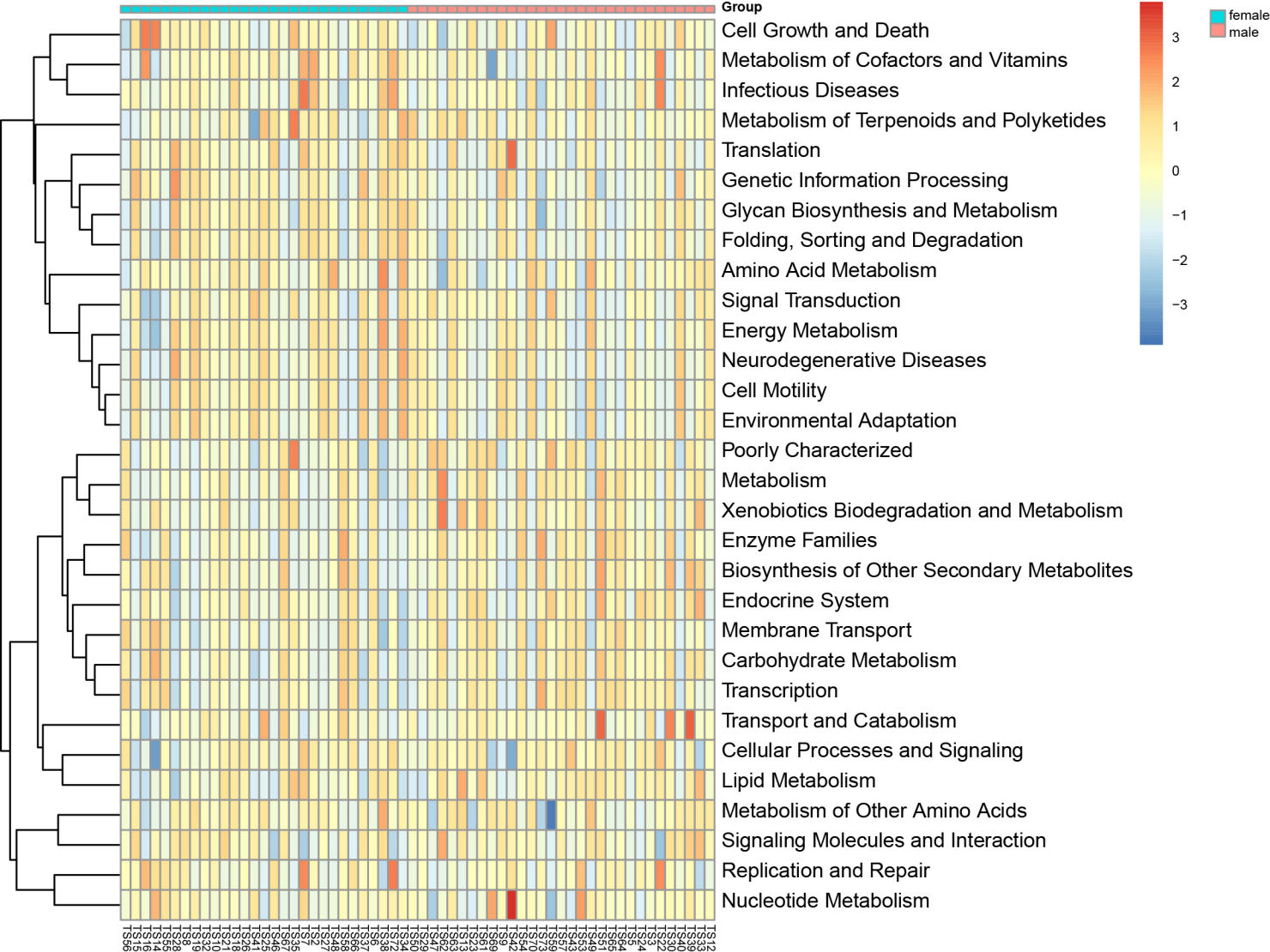

Supplement: Supplementary file 5 — Heatmap of the metabolic pathways results of gender groups (level 2). (PDF 1021 kb) [file 12866_2019_1581_MOESM5_ESM.pdf]

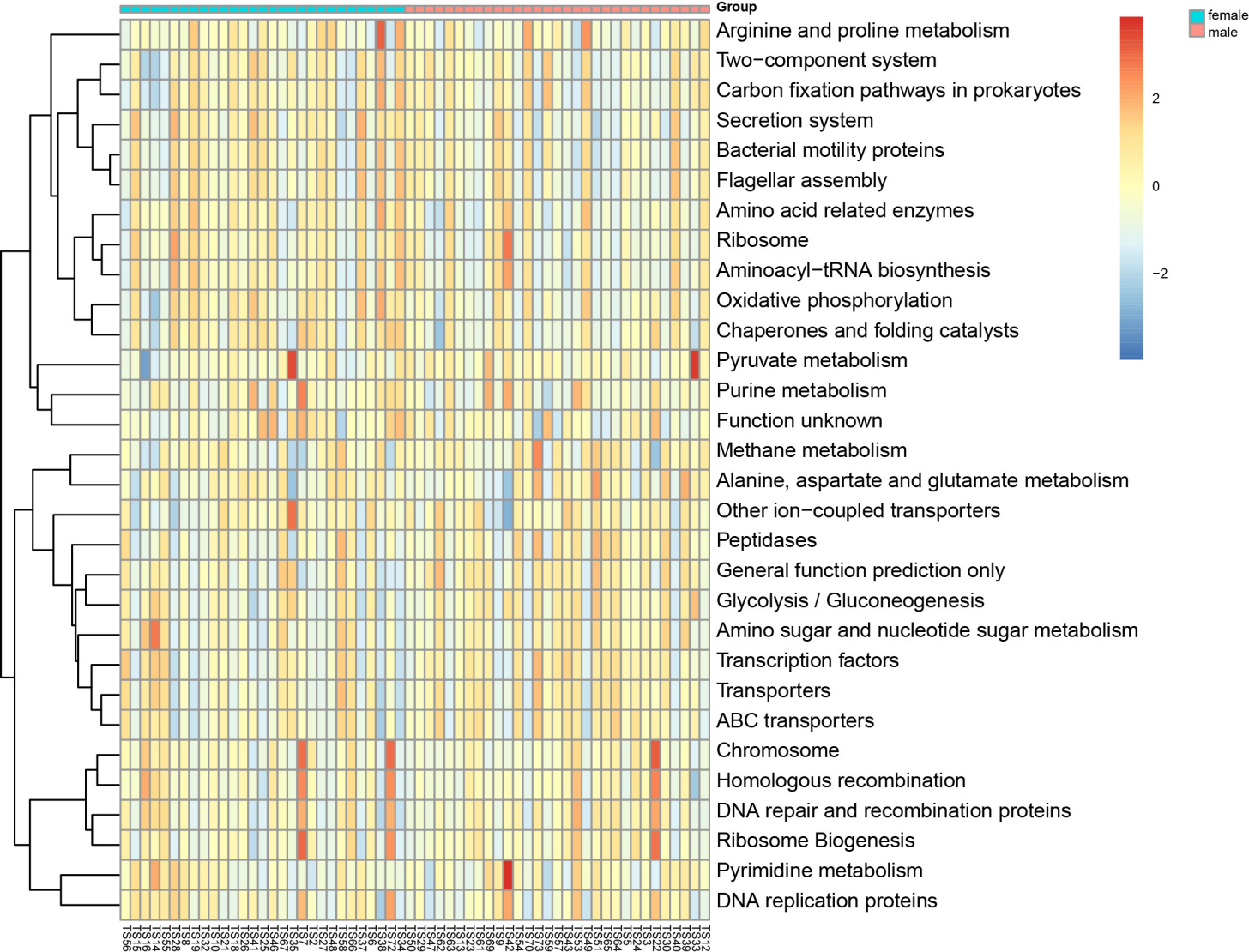

Supplement: Supplementary file 6 — Heatmap of the detailed annotation information for metabolic pathway among gender groups (level 3). (PDF 1016 kb) [file 12866_2019_1581_MOESM6_ESM.pdf]
