# Supplementary material for: The characteristics of gut microbiota and commensal Enterobacteriaceae isolates in tree shrew (Tupaia belangeri)
Source: BMC Microbiol. 2019 Sep 2;19:203. doi: 10.1186/s12866-019-1581-9 (PMC6721287; doi:10.1186/s12866-019-1581-9)
Supplement: Supplementary file 7 — The workflow for Enterobacteriaceae isolation and identification in this study. (PDF 1115 kb) [file 12866_2019_1581_MOESM7_ESM.pdf]

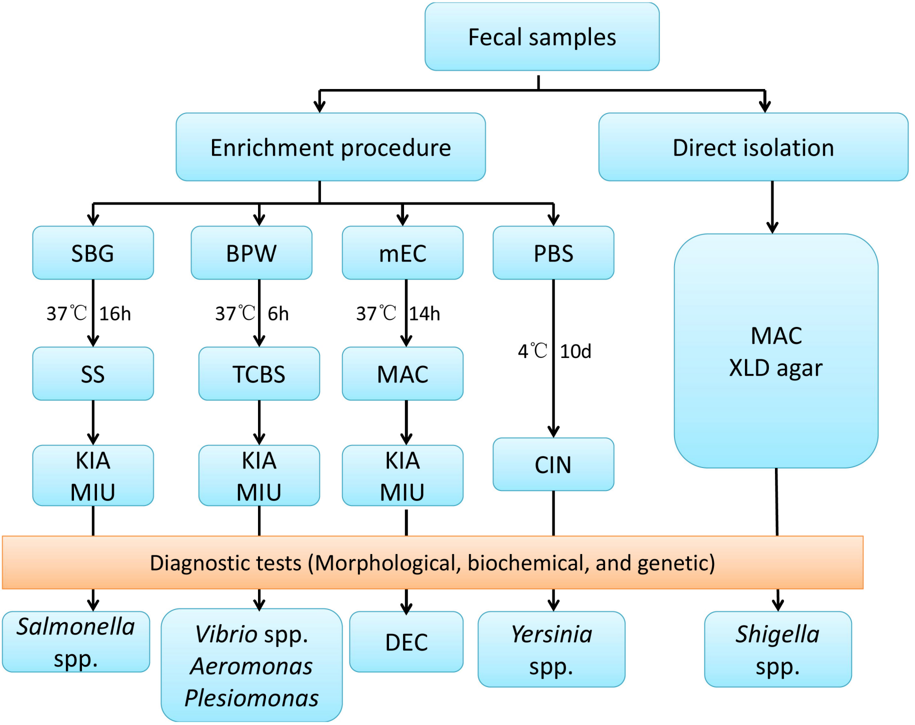

Abbreviation: SBG, selenite brilliant green sulfa enrichment; BPW, buffered peptone water; mEC, mE. Coli broth; PBS, phosphate buffered saline; SS, Salmonella Shigella agar; TCBS, thiosulfate citrate bile salts sucrose agar; CIN, Cefulodin Irgasan Novobiocin agar; MAC, MacConkey's agar; XLD, Xylose lysine deoxycholate agar; KIA, kligler iron agar; MIU, motility indole urea semisolid medium
