# Supplementary material for: The characteristics of gut microbiota and commensal Enterobacteriaceae isolates in tree shrew (Tupaia belangeri)
Source: BMC Microbiol. 2019 Sep 2;19:203. doi: 10.1186/s12866-019-1581-9 (PMC6721287; doi:10.1186/s12866-019-1581-9)
Supplement: Supplementary file 8 — The breakpoints of MIC values for Enterobacteriaceae bacteria in this study. (PDF 102 kb) [file 12866_2019_1581_MOESM8_ESM.pdf]

Additional file 8. The breakpoints of MIC values according to CLSI standard in this study

Antibiotics	MIC breakpoints (ug/ml)		
	Sensitive (S)	Intermediate (I)	Resistant (R)
Amoxicillin (AML)	≤8	16	≥32
Amoxicillin/Clavulanic acid (AMC)	≤8/4	16/8	≥32/16
Oxacillin (OX)	≤8	16	≥32
Cefotaxime (CTX)	≤1	2	≥4
Ceftazidime (CAZ)	≤4	8	≥16
Imipenem (IPM)	≤1	2	≥4
Meropenem (MEM)	≤1	2	≥4
Ciprofloxacin (CIP)	≤1	2	≥4
Gentamicin (CN)	≤4	8	≥16
Amikacin (AK)	≤16	32	≥64
Erythromycin (E)	≤2	4	≥8
Tetracycline (TE)	≤4	8	≥16
