# Supplementary material for: The characteristics of gut microbiota and commensal Enterobacteriaceae isolates in tree shrew (Tupaia belangeri)
Source: BMC Microbiol. 2019 Sep 2;19:203. doi: 10.1186/s12866-019-1581-9 (PMC6721287; doi:10.1186/s12866-019-1581-9)
Supplement: Supplementary file 9 — The PCR primers for different resistant genes in this study. (PDF 359 kb) [file 12866_2019_1581_MOESM9_ESM.pdf]

Additional file 9. The primers for antibiotic resistant-associated genes in this study

	Genes	Primers	Sequences (5'-3')	Annealing Temp (°C)	Fragment (bp)
β-lactamase genes	*bla*TEM	TEM-F	TCAACATTTCCGTGTCG	56	860
		TEM-R	CTGACAGTTACCAATGCTTA		
	*bla*SHV	SHV-F	ATGCGTTATATTCGCCTGTG	56	896
		SHV-R	AGATAAATCACCACAATGCGC		
	*bla*CTX-M-1	CTX-M-1F	CCGTTTCCGCTATTACAAACCG	56	944
		CTX-M-1R	GGCCCATGGTTAAAAAATCACTGC		
	*bla*CTX-M-2	CTX-M-2F	ATGATGACTCACAGCATTCG	56	833
		CTX-M-2R	TCCCGACGGCTTTCCGCGTT		
	*bla*CTX-M-8	CTX-M-8F	TTTGCCCGTGCGATTGG	50	368
		CTX-M-8R	CGACTTTCTGCCTTCTGCTCT		
	*bla*CTX-M-9	CTX-M-9F	ATGGTGACAAAGAGAGTGCA	50	870
		CTX-M-9R	CCCTTCGGCGATGATTCTC		
	*bla*CTX-M-10	CTX-M-10F	GCAGCACCAGTA AAGTGATGG	56	524
		CTX-M-10R	GCGATATCGTTGGTGGTACC		
	*bla*CTX-M-14	CTX-M-14F	GAGAGTGCAACGGATGATG	56	941
		CTX-M-14R	TGCGGCTGGGTAAAATAG		
AmpC genes	*bla*ACT	ACT-F	ATTCGTATGCTGGATCTCGCCACC	50	396
		ACT-R	CATGACCCAGTTCGCCATATCCTG		
	*bla*FOX	FOX-F	CACCACGAGAATAACC	50	1184
		FOX-R	GCCTTGAACTCGACCG		
	*bla*DHA	DHA-F	AACTTTCACAGGTGTGCTGGGT	60	405
		DHA-R	CCGTACGCATACTGGCTTTGC		

	*ba*CMY-G1	CMY-G1-F	GCTGACAGCCTCTTTCTCCAC	56	1082
		CMY-G1-R	CCTCGACACGGRCAGGGTTA		
	*ba*CMY-G2	CMY-G2-F	GGTCTGGCCCATGCAGGTGA	56	963
		CMY-G2-R	GGTCGAGCCGGTCTTGTTGA		
Carbapenem genes	*NDM1*	NDM1-F	GGTTTGGCGATCTGGTTTTC	52	621
		NDM1-R	CGGAATGGCTCATCACGATC		
	IMP	IMP-F	GGAATAGAGTGGCTTAAYTC	52	232
		IMP-R	TCGGTTTAAYAAAACAACCACC		
	VIM	VIM-F	GATGGTGTTTGGTCGCATA	52	390
		VIM-R	CGAATGCGCAGCACCAG		
	OXA-48	OXA-48-F	GCGTGGTTAAGGATGAACAC	52	438
		OXA-48-R	CATCAAGTTCAACCCAACCG		
	KPC	KPC-F	CGTCTAGTTCTGCTGTCTTG	52	798
		KPC-R	CTTGTCATCCTTGTTAGGCG		
Fluoroquinolone resistant-associated genes	*qnrA*	qnrA-F	ATTTCTCACGCCAGGATTTG	55	627
		qnrA-R	GATCGGCAAAGGTTAGGTCA		
	qnrB	qnrB-F	GATCGTGAAAGCCAGAAAGG	55	469
		qnrB-R	ACGATGCCTGGTAGTTGTCC		
	qnrC	qnrC-F	GGGTTGTACATTTATTGAATCG	55	307
		qnrC-R	CACCTACCCATTTATTTTCA		
	qnrD	qnrD-F	CGAGATCAATTTACGGGGAATA	55	533
		qnrD-R	AACAAGCTGAAGCGCCTG		
	qnrS	qnrS-F	ACGACATTCGTCAACTGCAA	55	417
		qnrS-R	TAAATTGGCACCCTGTAGGC		

	aac(6')-Ib-cr	aac(6')-Ib-cr-F	TTGCGATGCTCTATGAGTGGCTA	55	482
		aac(6')-Ib-cr-R	CTCGAATGCCTGGCGTGTTT		
	qepA	qepA-F	AACTGCTTGAGCCCGTAGAT	55	596
		qepA-R	GTCTACGCCATGGACCTCAC		
	gyrA	gyrA-F	CGACCTTGCGAGAGAAAT	55	626
		gyrA-R	GTTCCATCAGCCCTTCAA		
	parC	parC-F	TACGTCATCATGGACAGG	55	460
		parC-R	GCCACTTCACGCAGGTTG		
Aminoglycoside resistant-associated genes	*aacA4*	aacA4-F	ATGACTGA CATGACCTTGCG	55	540
		aacA4-R	TTAGGCATCACTGCGTGTTCG		
	aacC1	aacC1-F	ATGGGCATCATTCGCACATGTAGG	55	873
		aacC1-R	TTAGGTGGCGGTACTTGGGTC		
	aacC2	aacC2-F	ATGCATACGCGGAAGGCAATAAC	55	861
		aacC2-R	CTAACCGGAAGGCTCGCAAG		
	aadA1	aadA1-F	ATGAGGGAAGCGGTGATCG	55	792
		aadA1-R	TTATTTGCCGACTACCTTGGTG		
	aadB	aadB-F	ATGGACACAACGCAGGTCGC	55	534
		aadB-R	TTAGGCCGCATATCGCGACC		
	aphA6	aphA6-F	ATGGAATTGCCCAATATTATTC	55	781
		aphA6-R	TCAATTCAATTCATCAAGTTTTA		
	armA	armA-F	AGGTTGTTTCCATTTCTGAG	55	591
		armA-R	TCTCTTCATTCCCTTCTCC		
	rmtB	rmtB-F	CCCAAACAGACCGTAGAGGC	55	585
		rmtB-R	CTCAAACTCGGCGGGCAAGC		
Tetracycline	*tet(A)*	tet(A)- F	TTGGCATTCTGCATTCACTC	60	494

		tet(A)- R	GTATAGCTTGCCGGAAGTCG		
	tet(B)	tet(B)- F	CAGTGCTGTTGTTGTCATTAA	60	571
		tet(B)- R	GCTTGGAATACTGAGTGTAA		
	tet(E)	tet(E)- F	TATTAACGGGCTGGCATTTC	55	544
		tet(E)- R	AGCTGTCAGGTGGGTCAAAC		
	tet(M)	tet(M)- F	ACACGCCAGGACATATGGAT	55	536
		tet(M)- R	ATTTCCGCAAAGTTCAGACG		
	tet(30)	tet(30)- F	CCGTCATGCAATTTGTGTTC	55	550
		tet(30)- R	TAGAGCACCCAGATCGTTCC		
	Tet(O)	Tet(O)-F	GCGGTAATTATGGGAAACGA	55	550
		Tet(O)-R	TTTCCCGCTGTTCAGATTTC		
Erythromycin	*ereA*	ereA-F	AACACCCTGAACCCAAGGGACG	55	420
		ereA-R	CTTCACATCCGGATTCGCTCGA		
	ereB	ereB-F	AGAAATGGAGGTTCATACTTACCA	55	546
		ereB-R	CATATAATCATCACCAATGGCA		
	mphA	mphA-F	AACTGTACGCACTTGC	55	837
		mphA-R	GGTACTCTTCGTTACC		
	mefA	mefA-F	AGTATCATTAATCACTAGTGC	55	348
		mefA-R	TTCTTCTGGTACTAAAAGTGG		
